# Cultural adaptation and psychometric evaluation of the Swedish version of the Reproductive Concerns After Cancer (RCAC) scale

**DOI:** 10.1186/s12955-020-01520-y

**Published:** 2020-08-06

**Authors:** Poorna Anandavadivelan, Maria Wiklander, Lars E. Eriksson, Lena Wettergren, Claudia Lampic

**Affiliations:** 1 Department of Women’s and Children’s Health, Karolinska Institutet, Solna, Sweden; 2 Department of Neurobiology, Care Sciences and Society, Karolinska Institutet, Huddinge, Sweden; 3 Department of Learning, Informatics, Management and Ethics, Karolinska Institutet, Stockholm, Sweden; 4 School of Health Sciences, City, University of London, London, UK; 5 Department of Infectious Diseases, Karolinska University Hospital, Huddinge, Sweden; 6 Department of Public Health and Caring Sciences, Uppsala University, Uppsala, Sweden

**Keywords:** Breast neoplasms, Fertility distress, Psychometric evaluation, Reproductive health, Survivorship, Translation

## Abstract

**Background:**

Reproductive concerns are common among young cancer survivors and include worries related to different aspects of fertility and parenthood. The Reproductive Concerns After Cancer (RCAC) scale is an 18-item scale with six dimensions, developed to capture a variety of such concerns. The aim of the present study was to describe the cultural adaptation of the RCAC scale into Swedish and evaluate its psychometric properties among young women who have undergone treatment for cancer.

**Methods:**

The RCAC was forward translated from English into Swedish and assessed for cultural adaptation based on a two-panel approach followed by cognitive interviews with the target group. For the psychometric evaluation, a Swedish cohort of 181 female young adult breast cancer survivors completed a survey including the RCAC scale approximately 1.5 years post-diagnosis. Psychometric properties were examined by analyses of construct validity (confirmatory factor analysis and convergent validity), data quality (score distribution, floor and ceiling effects), reliability and known-groups validity.

**Results:**

The confirmatory factor analysis yielded an acceptable fit (RMSEA 0.08, SRMR 0.09, CFI 0.92). Convergent validity was demonstrated by a negative correlation of moderate size (− 0.36) between the RCAC total score and the emotional function scale of the EORTC QLQ-C30. Reliability measured with Revelle Ω total was satisfactory (0.73–0.92) for five of the dimensions, and poor for the dimension Becoming pregnant (Revelle Ω total = 0.60); Cronbach’s alpha showed a similar pattern. Known-groups validity was indicated by significant RCAC mean score differences (MD), reflecting more concerns among women with a certain (MD 4.56 [95% CI 3.13 to 5.99]) or uncertain (MD 3.41 [95% CI 1.68 to 5.14]) child wish compared to those with no wish for (additional) children.

**Conclusion:**

The translation and cultural adaptation of the Swedish RCAC has resulted in a scale demonstrating construct and known-groups validity, and satisfactory reliability for five of six dimensions. The dimension Becoming pregnant showed non-optimal internal consistency and should undergo further evaluation. The Swedish RCAC is recommended to be used in research settings for measurement of concerns related to fertility and parenthood in young women with cancer.

## Background

A large number of individuals in the age bracket 18–39, defined as young adults [1], are diagnosed with cancer worldwide. In the United States alone, around 60,000 young adults are diagnosed with cancer yearly [2] and, correspondingly in Sweden, around 2000 young adults face a cancer diagnosis every year [3]. The cancer itself, or being exposed to surgical and/or gonadotoxic treatments may result in temporary or permanent infertility, or sub-fertility in young adult survivors [4], many of whom may not have had the possibility to start or complete their family prior to their cancer diagnosis. Young adult cancer survivors report a number of reproductive concerns after treatment, including worry regarding the ability to have children in the future, fears about recurrence, their child’s health and their own health [5, 6]. Among female cancer survivors, a wish for (additional) children has been shown to be associated with more worries about infertility [7]. Furthermore, higher levels of reproductive concerns have been found to be associated with poorer quality of life and depression [8].

To capture the full range of reproductive concerns following cancer the multidimensional Reproductive Concerns After Cancer (RCAC) scale was developed for use in young adult female survivors [7]. The scale has shown satisfactory construct validity and internal consistency among women aged 18–44 treated for different types of cancer in the United States [7, 9]. Outside the US context, the RCAC scale has been translated and culturally adapted into Chinese [10] and Portuguese [11], demonstrating suitable psychometric properties for evaluation of reproductive concerns in young patients with cancer.

The aim of the present study was to describe the translation and cultural adaptation of the RCAC scale into Swedish and to evaluate its psychometric properties among young women with breast cancer, using data collected in an earlier study [12].

## Methods

### The RCAC scale

The RCAC scale consists of 18 items that constitute six dimensions: Fertility potential, Partner disclosure, Child’s health, Personal health, Acceptance, and Becoming pregnant. Each dimension has three items with responses scored on a five-point scale (ranging from 1 = Strongly disagree to 5 = Strongly agree), with higher scores indicating higher levels of reproductive concerns [7]. The psychometric properties of the original scale in English showed that the internal consistency of the total RCAC scale was good (α =0.82), and good or acceptable for the six sub-scales (α = 0.78–0.86) [7]. Known-groups validity was demonstrated by significantly higher mean scores among women who wanted to have children compared to those who did not, and among those for whom having a biological child was very important compared to those for whom it was less important [7]. Total RCAC scores were positively associated with depression and negatively associated with social support and satisfaction with life.

### Swedish translation and cultural adaptation of the Swedish RCAC scale

For the purpose of use in a study from our research group [12], the original RCAC scale was translated and culturally adapted to be able to measure reproductive concerns in a Swedish context. To achieve this, the scale was forward translated into Swedish based on a dual panel approach and further assessed for cultural adaptation by lay panel, patient/target group assessment and cognitive interviews [13]. The process of translation and cultural adaptation was coordinated by the same coordinator to ensure none of the parameters were neglected and to maintain the quality of the adaptation.

#### Forward translation by bilingual expert panel

The RCAC scale was translated from English to Swedish by two researchers who were native Swedish speakers and well-versed in English. The first translator (T1) had extensive research knowledge in the field of fertility and cancer. The second translator (T2) coordinated the translation and was experienced in instrument translation and adaptation, but was not knowledgeable in the research fields of fertility and cancer. Both researchers had broad experience of clinical work with diverse patient groups. Following individually performed translations of the scale, T1 and T2 discussed discrepancies between the two versions. As a next step, they consulted the principal investigator of the original English version of the RCAC to discuss and clarify the intended conceptual meaning of the scale and specific items. Subsequently, the two translators (T1 and T2) and two additional experts in the field of psychosocial oncology (native Swedish speakers) discussed the translation in a panel meeting. As a result, a consensus version of the Swedish version of the RCAC was created. Cultural adaptation included changing the use of “spouse/partner” in the three items of the dimension Partner disclosure to “partner”, as this was deemed more appropriate for use in the Swedish context where it is common for two adults to live together without being married.

#### Lay panel assessment

For evaluation of the translated version of the scale, two lay panels were recruited through personal contacts and local advertisements. The panel members were 3 women and 4 men between 18 and 41 years old; three had secondary education and four had higher education. Two had children and none of the panel members had been diagnosed with cancer. Panel members were compensated with cinema tickets for their participation. The lay panels were only provided with the Swedish version of the scale, as suggested [13]. The lay panel members were instructed to go through all parts of the RCAC scale, including instructions, items and response options. Everyone read each item, then discussed how they perceived the issue and whether there were any alternative ways of phrasing the question. Based on the assessment by the lay panels, minor changes in wording were made. The main role of the lay panels was to produce a version that was easy to understand for the average Swedish speaking person. The lay panel assessment was led by the same coordinator as for the expert panel.

#### Patient/target group assessment

The patient/target group included 5 women and 3 men (aged 20–41) who had been treated for cancer. The target group members came from different geographical areas in Sweden and all had secondary or higher education. They evaluated the translated RCAC scale for face validity i.e., if the items and response alternatives were relevant and acceptable. Some concerns were expressed regarding the suitability and relevance of the scale for patients in their late teens, which led us to conducting cognitive interviews as described below. The target group members were compensated for their travel costs and time spent.

#### Cognitive interviews

Cognitive interviews were performed individually with 3 young individuals (1 female aged 18 years and 2 males aged 17 years) currently being treated for cancer. The participants completed the Swedish RCAC scale and were then interviewed on their experience of responding to the items. We used a flexible approach as suggested earlier [14]. In brief, respondents were asked to *think aloud* and share thoughts, perceptions and opinions that came up when answering the questions. The interviewer probed every issue that was mentioned to capture difficulties related to the items of the RCAC. We asked if items were difficult to understand (wording, knowledge requested and information given), included inappropriate assumptions, were too sensitive (content, wording and social acceptability), as well as if the response categories were adequate.

### Psychometric evaluation

#### Participants and procedure

A detailed description of the study participants and procedure is presented elsewhere [12]. Briefly, a sample of 301 women consecutively diagnosed with invasive breast cancer (all stages except in-situ and very few with distant metastasis) at age 18–39 years was identified from the Swedish National Quality Register for Breast Cancer. Data collection was conducted by means of a comprehensive postal survey approximately 1.5 years post-diagnosis. Ethical approval for the study was obtained from the Regional Ethical Review Board in Stockholm, Sweden (Ref No: 20131746–31/4).

#### Additional measures

The survey completed by the participants comprised several patient-reported outcome measures. For the purpose of the psychometric evaluation of the Swedish version of the RCAC, the Emotional Function (EF) scale of the EORTC QLQ-C30 version 3.0 [15] and a study-specific item regarding current wish to have (additional) children (response alternatives: Yes, Uncertain, No) were used from the survey.

### Statistical analysis

Statistical analysis was performed using SPSS statistics for Windows, version 24 (IBM Corp., Armonk, N.Y., USA) and IBM® SPSS® Amos, version 25 and R version 4.0.0 (The R Foundation for Statistical Computing) with the additional R package ‘userfriendlyscience’ [16]. For all statistical tests, the level of statistical significance was defined as *p* < 0.05.

Construct validity was ascertained using Confirmatory factor analysis (CFA) and convergent validity. CFA with maximum likelihood estimation was performed to determine the adequacy of the original six-factor structure of the RCAC on our sample data [7]. Four participants were excluded from the CFA due to not having completed the RCAC scale (*n* = 3) or missing values for 2 items in one dimension (*n* = 1). Missing values for single items (*n* = 15) were handled by imputing the mean of the other two items of the same individual in the same dimension. Standardized factor loadings and model fit were determined from the CFA. Standardized factor loadings of ≥ 0.4 were considered as acceptable as determined from the CFA [17]. Model fit was estimated by two absolute indices of overall model fit, Root mean square error of approximation (RMSEA) and Standardized root mean residual (SRMR), and one relative index of model fit compared to the null model, Comparative fit index (CFI). The acceptable thresholds for these indices were defined as RMSEA: 0.05–0.08, SRMR: < 0.10 and CFI: > 0.90 according to Kline’s [18] guidelines. The model chi-square (*χ*^2^), degrees of freedom (d.f.) and associated *p* value were reported, but were not considered as an indicator of model fit owing to their restrictiveness by being sensitive to sample size [19]. Additionally, the relative/normed *χ*^2^ ratio (*χ*^2^/d.f.) was also reported as recommended, and a cut-off value of < 5 was regarded as good [20]. Convergent validity was assessed by calculating the Pearson correlation coefficient for the mean scores of the Swedish RCAC scale and the EF scale of the EORTC QLQ-C30. The EF scale covers both symptoms of anxiety and depression and is a robust measure of emotional distress in patients with cancer [21]. The construct represented by the EF was thus expected to be related to RCAC scores although it is distinct from that of reproductive concerns. We hypothesized that RCAC total scores would show a negative association of moderate size with EF (higher scores of the scale indicate better EF). Correlation coefficients of 0.10–0.29, 0.30–0.49 and 0.50 and above were interpreted as small, moderate and large, respectively [22, 23].

Data quality was assessed by examination of missing values, mean scores, standard deviations (SD), and floor and ceiling effects. Floor and ceiling effects were considered present if > 15% rated at the lowest (floor) and highest (ceiling) scores [24].

Reliability was assessed by means of the internal consistency of the six dimensions using Cronbach’s α as well as Ω total and Revelle Ω total [25]; values of ≥ 0.70 were considered acceptable [26].

Known-groups validity was assessed by comparing groups of women who differed in their reported wish for children or additional children. It was hypothesised that women with a wish for children would report higher levels of reproductive concerns than those without such a wish, in line with previous reports [7, 27]. A one-way ANOVA with post-hoc comparisons using the Tukey test was conducted to compare the mean scores of the Swedish RCAC scale of the three groups of women who reported a certain, uncertain or no wish for (additional) children respectively.

## Results

### Sample characteristics

Of 301 eligible women, 181 completed the survey (response rate 60%). Demographic and clinical characteristics of the participants are presented in detail elsewhere [12]. Briefly, mean age was 36.5 (SD 4.1, range 23–42), a majority had children (77%) and were in a current partner relationship (87%). A wish for (additional) children was expressed by 36% of the women, 20% were uncertain and 44% had no current child wish.

### Construct validity

#### Confirmatory factor analyses

**Fig. 1:**
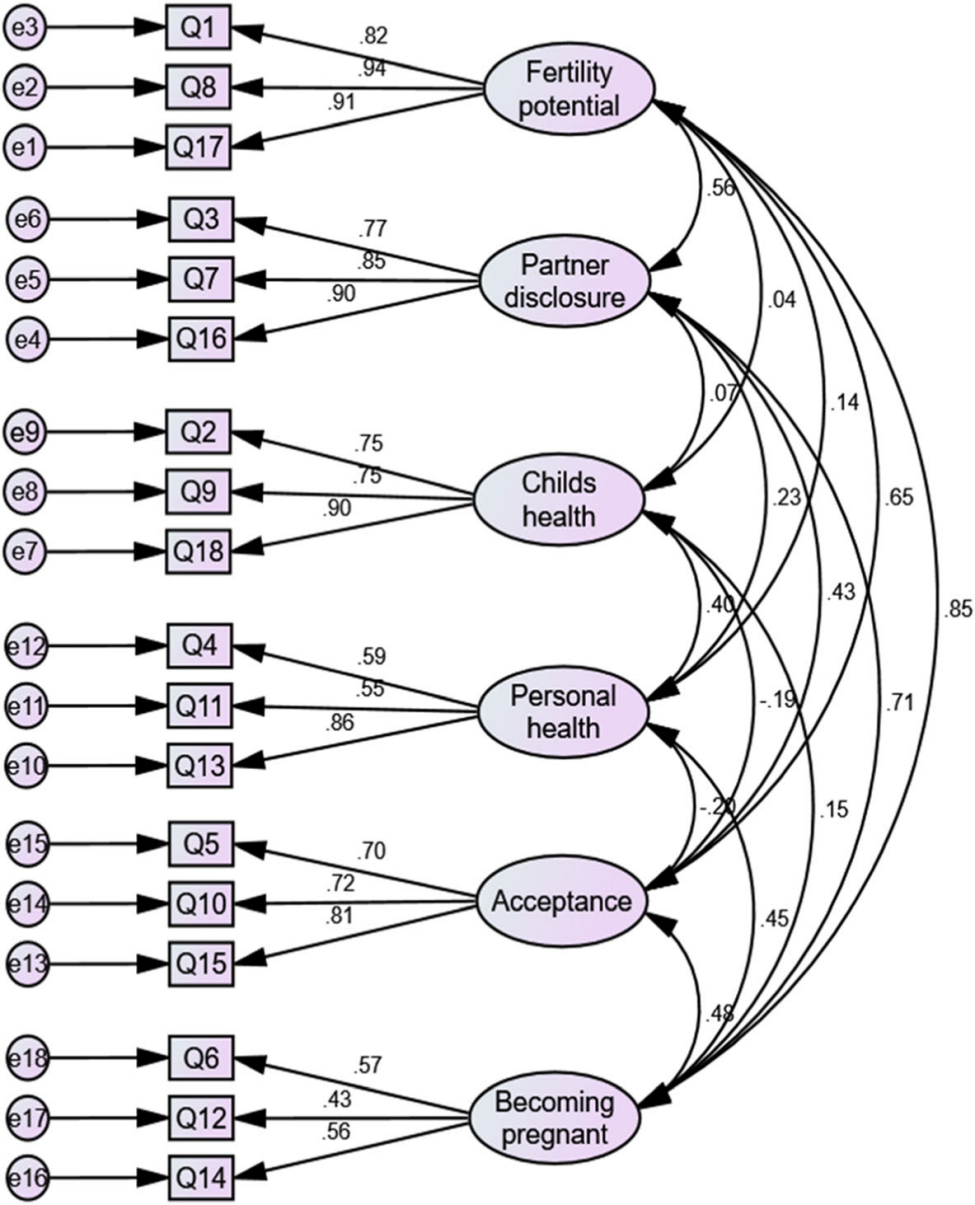
CONFIRMATORY Factor Analysis model of the Swedish RCAC scale on a sample of 177 breast cancer survivors

The CFA with maximum likelihood estimation was performed on the responses of 177 participants with complete data after imputation of missing values. The CFA provided a relative/normed *χ*^2^ value of 2.06 (*χ*^2^ = 246.543; degrees of freedom = 120; *p* < 0.001). The CFA provided an RMSEA of 0.08 (90% CI 0.06–0.09), an SRMR value of 0.09 and a CFI of 0.92, indicating an acceptable fit. Standardized factor loading estimates ranged from 0.43 to 0.94 and were above the acceptable factor loading cut-off (Fig. 1). Standardized factor loadings were statistically significant (*p* < 0.01) for all six of the dimensions (data not shown).

#### Convergent validity

The Swedish RCAC total scale score and the EF scale of the EORTC QLQ-C30 showed a negative correlation with a correlation coefficient of moderate size (*r* − 0.36).

### Data quality

Descriptive statistics of the RCAC dimensions are presented in Table 1. There were few missing responses (< 1%/ item). Item means and standard deviations within dimensions were roughly equivalent with one exception, means of the items of the Personal health dimension had a slightly wider range (2.67 to 4.12); all response alternatives were used for all items. A floor effect above the customary cut-off of 15% was observed in the dimensions Fertility potential (21%), Partner disclosure (34.3%) and Becoming pregnant (17.1%). A ceiling effect (> 15%) was detected in the dimension Child’s health (17.7%).

**Table 1:** Descriptive statistics and internal reliability of the six RCAC dimensions

Dimension	Mean (SD)	Range of item means (SD)	Floor/ceiling effects (%)	α (95% CI)	Ω Total (95% CI)	Revelle Ω Total
Fertility Potential	2.75 (1.32)	2.67–2.91 (1.36–1.46)	21.0/7.2	0.92 (0.89–0.94)	0.92 (0.90–0.94)	0.92
Partner disclosure	2.17 (1.12)	2.07–2.28 (1.22–1.27)	34.3/2.2	0.88 (0.84–0.91)	0.88 (0.85–0.91)	0.88
Child’s health	3.58 (1.21)	3.48–3.69 (1.32–1.40)	6.1/17.7	0.84 (0.80–0.88)	0.85 (0.81–0.89)	0.85
Personal health	3.27 (1.06)	2.76–4.12 (1.24–1.41)	5.0/7.7	0.68 (0.60–0.76)	0.73 (0.66–0.80)	0.73
Acceptance	2.44 (1.09)	2.33–2.57 (1.22–1.38)	13.3/3.9	0.78 (0.72–0.84)	0.78 (0.73–0.84)	0.79
Becoming pregnant	2.41 (0.90)	2.33–2.53 (1.19–1.27)	17.1/2.2	0.54 (0.42–0.66)	0.58 (0.47–0.68)	0.60

### Reliability

The Ω total and Revelle Ω total were above considered acceptable (≥ 0.70) for five of the six dimensions, and poor for the dimension Becoming pregnant (Ω total 0.58, Revelle Ω total 0.60; 95% CI 0.42–0.66), see Table 1; the Cronbach’s alpha showed a similar pattern.

### Known-groups validity

The results for the known-groups validity investigation showed a significant difference in RCAC total mean scores between the three groups based on self-reported child wish (F = 29.54, *p* < 0.001). Statistically significant group differences were found between, on the one hand, women with a wish for (additional) children and, on the other hand, women who were uncertain if they wanted children (MD 3.41; 95% CI 1.68–5.14) and women who had no child wish (MD 4.56; 95% CI 3.13–5.99). No statistically significant difference in RCAC scores was observed between the latter two groups (uncertain vs no child wish) (MD 1.15; 95% CI − 0.53 to 2.82).

## Discussion

In the present study, the psychometric properties of the Swedish RCAC scale were evaluated in young survivors of breast cancer while retaining the same factor structure as the original version of the scale. The translated and culturally adapted scale demonstrated face validity in evaluation with experts and persons from the target group. The CFA provided an acceptable fit and all dimensions except Becoming pregnant displayed satisfactory reliability. Convergent validity was demonstrated by a moderate negative association with emotional function, and known-groups validity was shown by statistically significant differences in RCAC scores between groups with and without a wish for (additional) children.

The present results demonstrated reasonable evidence to support the six-factor structure of the Swedish RCAC. Although differences between the strength of model fit indices were observed for the Swedish version of the RCAC, the Chinese RCAC scale [10] and the re-validated RCAC scale [9], an overall comparison of construct validity showed compliance with the six-factor structure of the original RCAC [7] consistently across all studies. However, in a recent study describing the Portuguese RCAC, explanatory factor analysis led to a five-factor structure [11]. In the present study the RMSEA was within an acceptable level of error of approximation, the SRMR value was slightly outside the suggested range although not pointing to a poor fit, and the CFI indicated a reasonably good fit [18]. It must be noted that while some fit indices may point to a well-fitting model, parts of the fit indices may show non-optical figures [19]. This, together with the fact that there are multiple and not rigid guidelines available for ”acceptable” model fit [20], makes us conclude that our analyses overall show that the model has an acceptable fit. However, even though the sample size of our study met the rule of thumb for CFA given a non-complex model [18], future studies with larger samples are needed to validate our findings for the Swedish RCAC.

As expected, the Swedish RCAC demonstrated a negative moderate association with the EF subscale of the EORTC QLQ-C30. This finding was consistent with previous studies showing that high levels of the RCAC are associated with lower levels of emotional function [11], social support and satisfaction with life [25]. These results are also in line with previous reports that higher RCAC scores are associated with anxiety and depression [7, 10, 11]. The present and previous results [7, 10, 11] indicate that reproductive concerns, as measured by the RCAC, represent a construct that is related to but distinct from anxiety and depression.

The differences with regard to the strength of the model fit indices and convergent validity may be attributed to differences in the sampling techniques between the studies. In the studies describing the original RCAC, participants were recruited via social media outreach and fertility preservation programs [7, 9] and the study of the Portuguese RCAC used convenience sampling [11]. This may have resulted in an overrepresentation of female cancer survivors interested in having children, as also pointed out by some of the researchers. While the population-based sample in our study may be regarded as more representative for survivors of breast cancer owing to a lower risk of selection bias, the socio-demographics of the sample differed from those in previous studies. In comparison to the studies mentioned above [7, 11], larger proportions of women in the present study were currently in a partner relationship, already had children and had no wish for (additional) children. Hence it is not surprising that many participants chose the lowest possible scores for the dimensions Partner disclosure, Fertility potential and Becoming pregnant, resulting in floor effects. Similarly, a ceiling effect was seen for the subscale Child’s health that focuses on concerns related to a potential hereditary risk of cancer for offspring. This finding may be attributed to the fact that the present sample consisted of women diagnosed with breast cancer under the age of 40, who are at higher risk of hereditary cancer gene mutations [28]. Thus, in comparison to previous studies of the RCAC that included lower percentages of breast cancer survivors [7, 9, 11], more women in our cohort may have had reason to be concerned about a genetic cancer risk for their children.

Known-groups validity of the Swedish RCAC was shown by significantly higher levels of reproductive concerns among women who wanted to have children compared to those without a child wish, as hypothesised. These results are in line with those reported for the original RCAC [7] and recently for the Portuguese version of the RCAC [11].

Reliability, exhibited by Ω, was above the acceptable range in five of the six dimensions. Only the Becoming pregnant dimension was below the acceptable limit in our study. These results are similar to the results of the psychometric validation of the original RCAC scale [9]. In the explanatory factor analysis of the Portuguese version of the scale the dimension Becoming pregnant was not replicated as per the six-factor model, but its items loaded on two different factors [11]. These results may suggest that the items in Becoming pregnant are likely more difficult to comprehend and/or that this dimension does not capture an equally clear construct as the other dimensions. This also emphasises the need to consider testing other psychometric validation methods besides factor analysis, such as item response theory (IRT), to analyse the properties of the RCAC instrument in future studies.

A particular strength of the design of the present study is the nationwide population-based sample of young adult breast cancer survivors identified from a valid national quality register, which minimised selection bias related to reproductive concerns. Also, the translation of the Swedish version of the RCAC scale was conducted based on a dual panel approach [13]. Although back translation is still considered the gold standard, dual panel methodology has been shown to perform equally well and to also have specific linguistic advantages [13, 29].

## Conclusions

The translation and cultural adaptation of the Swedish RCAC has resulted in a scale demonstrating construct and known-groups validity, and satisfactory reliability for five of six dimensions. In line with previous studies the dimension Becoming pregnant showed non-optimal internal consistency and the dimension is recommended to undergo further evaluation. Based on the overall results, with the exception of the Becoming pregnant dimension, the Swedish RCAC is recommended to be used in research settings for measurement of concerns related to fertility and parenthood in young women with cancer. The knowledge generated from such studies may be useful for identification of vulnerable groups throughout the cancer survivorship trajectory.

## Data Availability

The datasets generated and/or analysed during the current study are available from the principal investigators of the study on reasonable request.

## Abbreviations

ANOVA: Analysis of variance
CFA: Confirmatory factor analysis
CFI: Comparative fit index
CI: Confidence interval
EORTC: European Organization for Research and Treatment of Cancer
MD: Mean score differences
QLQ-C30: Quality of Life Questionnaire-C30
RCAC: Reproductive Concerns After Cancer Scale
RMSEA: Root mean square error of approximation
SRMR: Standardized root mean residual
